# Efficacy of Fetal Ear Length as a Prenatal Marker of Chromosomal Anomalies: A Prospective, Multicenter Cohort Study in a Southern European Population

**DOI:** 10.1002/jcu.70040

**Published:** 2025-08-14

**Authors:** Elisabet Baldrich, Montse Comas, Alicia Maldonado, Marcos Blasco, Josep‐Maria Manresa‐Domínguez, Maria Iniguez‐Cruz, Antoni Borrell

**Affiliations:** ^1^ Obstetrics and Gynecology Primary Care Center (ASSIR) Sabadell Institut Catala de la Salut Barcelona Spain; ^2^ Research Group on Sexual and Reproductive Healthcare (GRASSIR) (2021‐SGR‐793) Barcelona Spain; ^3^ Facultat de Medicina i Ciències de la Salut, Universitat de Barcelona (UB) Barcelona Catalonia Spain; ^4^ Gynecology and Obstetrics Service, Parc Tauli University Hospital Parc Taulí Research and Innovation Institute (I3PT‐CERCA), Universitat Autònoma de Barcelona Sabadell Catalonia Spain; ^5^ Unitat de Suport a la Recerca Metropolitana Nord Fundació Institut Universitari per a la recerca a l'Atenció Primària de Salut Jordi Gol i Gurina (IDIAPJGol) Mataró Catalonia Spain; ^6^ BCNatal Barcelona Centre for Maternal‐Fetal and Neonatal Medicine (BCNatal), Hospital Clínic Barcelona Universitat de Barcelona Barcelona Catalonia Spain

**Keywords:** chromosomal anomaly, cohort study, ear length, fetal ultrasonography

## Abstract

**Objective:**

To develop a nomogram of fetal ear length (FEL) by gestational age in a healthy pregnant Southern European population and assess its potential as a prenatal ultrasound marker of chromosomal anomalies in this demographic.

**Methods:**

This prospective, multicenter cohort study included low‐risk pregnancies from 11 + 2 to 34 + 6 gestational age. A nomogram was constructed based on gestational age for healthy fetuses with normal perinatal outcomes. Intraobserver and interobserver reliability were evaluated. To assess the efficacy of FEL as a marker of chromosomal anomalies, a multivariate logistic regression analysis was performed; sensitivity and specificity were calculated.

**Results:**

A total of 1923 FEL measurements were obtained from 1331 singleton pregnancies. Using data from healthy fetuses, a nomogram was constructed through linear regression analysis. Measurement feasibility was excellent, with intra‐ and interobserver correlation coefficients of 0.996 (95% confidence interval [CI]: 0.995–0.997) and 0.998 (95% CI: 0.978–0.999), respectively. FEL achieved a sensitivity of 81.8% and a specificity of 49.8% in detecting chromosomal anomalies. Multivariate logistic regression indicated that FEL ≤ 5th percentile significantly increased the likelihood of detecting chromosomal anomalies (odds ratio = 3.11); although the wide 95% CI (1.92–10.7) suggests a cautious interpretation of this finding.

**Conclusions:**

While FEL demonstrates potential as a prenatal marker of chromosomal anomalies, its clinical utility remains limited due to moderate sensitivity and specificity. Further studies are warranted to refine its diagnostic value in routine screening practices.

## Graphical abstract

Fetal ear length (FEL) correlates with gestational age and may help detect chromosomal anomalies. This study developed a nomogram for a Southern European population, showing high measurement reliability. While FEL ≤ 5th percentile increased anomaly risk, its moderate sensitivity and specificity limit clinical utility.

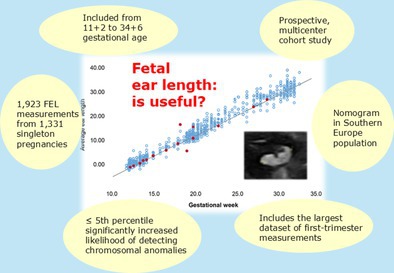

## 1 Introduction

Chromosomal anomalies occur in approximately 1% of newborns. In Catalonia, First‐trimester Combined Screening (FTS) has been routinely offered for prenatal detection of chromosomal anomalies since 2018. This screening test is available to all pregnant individuals, regardless of maternal age, provided that high‐risk factors based on personal or familial history have been excluded. FTS achieves a 90% detection rate for trisomy 21 with a 4% false‐positive rate. In late booking pregnancies, at 14 weeks or later, second‐trimester biochemical screening (quadruple test) is recommended, which has a 75% detection rate (Generalitat de Catalunya 2018a, 2018b, 2022).

More recently, cell‐free DNA (cfDNA) testing has emerged as a superior screening method, offering higher detection rates: 99% for Down syndrome, 97% for trisomy 18, and 92% for trisomy 13. In Catalonia's public health system, cfDNA testing has been available since 2021 for high‐risk (1/10–1/250) and intermediate‐risk (1/251–1/1100) cases following FTS (Generalitat de Catalunya 2018a, 2018b).

Certain ultrasound markers have been evaluated as secondary aneuploidy indicators to improve the screening effectiveness of FTS or the quadruple test. For instance, nuchal translucency—already incorporated into the FTS—has been supplemented with markers such as the nasal bone and ductus venosus flow. Studies suggest that incorporating these markers enhances trisomy 21 detection rates; however, they have not been systematically integrated into routine FTS (Borrell i and Sabrià 2023; Nicolaides 2011; Illa et al. 2013; Agathokleous et al. 2013; Borrell et al. 2009; Bilardo et al. 2023).

Children with chromosomal anomalies frequently exhibit structural anomalies in the shape, size, or positioning of their ears. Dysmorphic, low‐set, and short ear length are commonly associated with genetic syndromes, including Down syndrome, Turner syndrome, and Edwards syndrome (Aase et al. 1973; Thelander and Pryor 1966; Sivan et al. 1983; Nuñez‐Castruita and López‐Serna 2018; Allanson et al. 1993). These ear characteristics serve as potential clinical markers during pediatric evaluations and prenatal ultrasound screenings. However, fetal ear assessment has not been widely adopted as a first or second‐trimester genetic marker, primarily due to limited research and controversial findings.

The aims of this study were as follows: (a) to develop a fetal ear length (FEL) nomogram for our population between 11 + 2 and 34 + 6 gestational age; (b) to assess intra‐ and interobserver measurement reliability; and (c) to validate the diagnostic performance of FEL in the detection of chromosomal anomalies.

## 2 Methods

This was a multicenter, prospective cohort study conducted to evaluate the potential of FEL as a prenatal ultrasound marker of chromosomal anomalies. Between September 2022 and June 2024, pregnant women undergoing ultrasound examinations between 11 + 2 and 34 + 6 weeks of gestation at two centers in Sabadell—Sabadell Sexual and Reproductive Health Care Center (ASSIR) and Parc Taulí University Hospital—were invited to participate and provide informed consent. Pregnancies identified as high risk for fetal chromosomal or structural anomalies were referred to Parc Taulí University Hospital for invasive prenatal diagnostic testing.

The study protocol was reviewed and approved by the Research Ethics Committees of IDIAP Jordi Gol (22/040‐P) at the Catalan Institute of Health and Parc Taulí University Hospital (2022/6059), ensuring adherence to ethical standards for prenatal research.

Gestational age was determined in the first trimester using crown‐rump length (CRL) measurements between 11 + 2 and 13 + 6 weeks or biparietal diameter (BPD) measurements between 14 + 0 and 23 + 6 weeks, based on the gestational age calculator from the Hospital Clínic de Barcelona (Fetal I+D Education Barcelona 2023).

First‐trimester ultrasound assessments included measurements of CRL, BPD, and nuchal translucency (NT) (Bilardo et al. 2023). During the second trimester, BPD, head circumference (HC), abdominal circumference (AC), and femur length (FL) measurements were recorded, while third‐trimester data incorporated estimated fetal weight and its percentile. Comprehensive fetal anatomical evaluations were performed to detect structural anomalies and identify ultrasound markers.

Ultrasound markers evaluated in the first trimester included NT thickness, nasal bone (NB) length, ductus venosus (DV) flow, and tricuspid flow. In the second trimester, nine markers were assessed: ventriculomegaly, increased nuchal fold thickness, absent or hypoplastic nasal bone, intracardiac echogenic focus, aberrant right subclavian artery, echogenic bowel, mild hydronephrosis, short femur, and short humerus (Generalitat de Catalunya 2018a; Hospital Clínic|Hospital Sant Joan de Déu|Universitat delona 2023).

**FIGURE 1:**
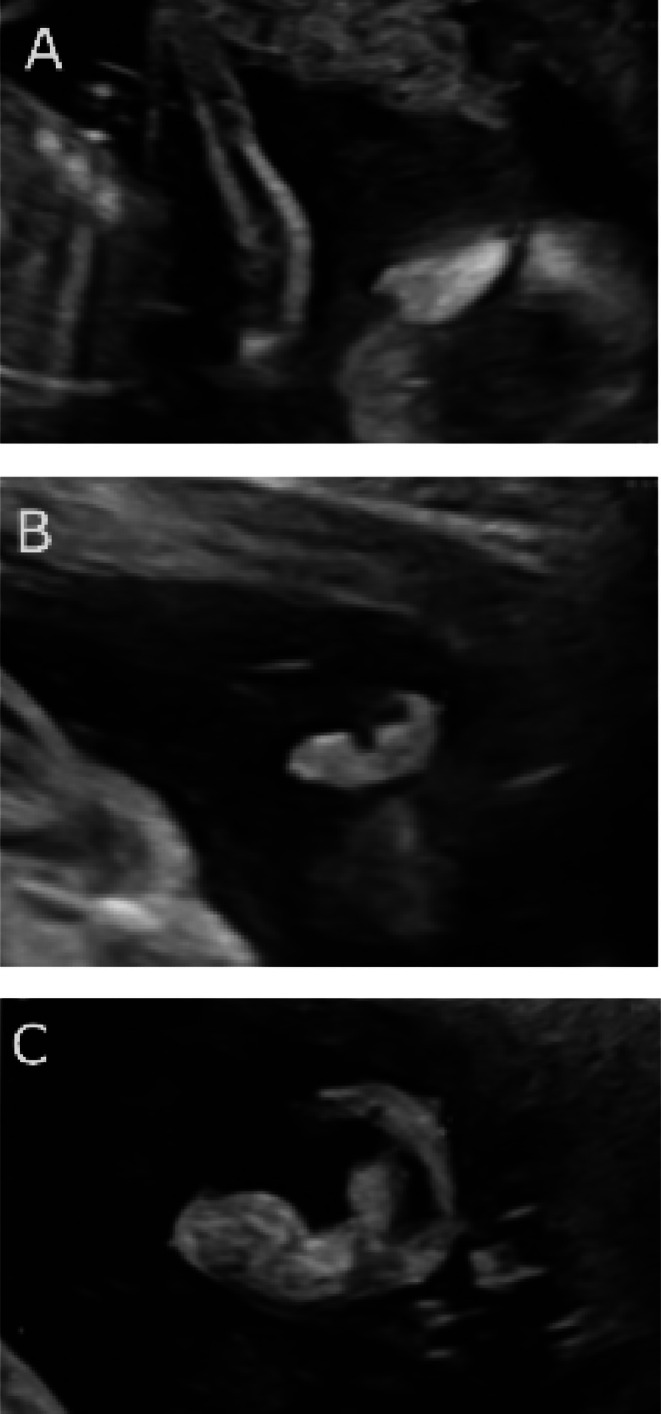
2D abdominal scan in a parasagittal view for fetal ear length (FEL) measurement. (A) 13‐week fetus; (B) 20‐week fetus; (C) 30‐week fetus.

Fetal ultrasound examinations were conducted by seven sonologists specializing in prenatal imaging—five at ASSIR Sabadell and two at Parc Taulí University Hospital. All scans utilized 2D imaging performed with Voluson S8 (General Electric) and Canon Aplio i700 ultrasound systems equipped with convex abdominal probes (2–7 MHz) or vaginal probes (6 MHz). FEL was measured in a parasagittal view of the fetal head as the maximum distance from the superior aspect of the helix to the inferior border of the earlobe for each ear (Figure 1). If measurements from both ears were available, the mean FEL was calculated; otherwise, a single measurement was recorded.

Maternal and pregnancy characteristics were summarized using absolute and relative frequencies for qualitative data. Continuous variables were expressed as the mean and standard deviation or median and interquartile range (Q1–Q3), depending on data distribution. For all pregnancies, the type and results of aneuploidy screening, genetic diagnostic tests (including karyotype, array‐comparative genomic hybridization (CGH), exome sequencing, and molecular studies), and pregnancy outcomes were recorded. The number of cases in which both ears, one ear, or neither could be assessed, along with the average examination time, were also documented.

The FEL nomogram by gestational age was constructed using data from healthy fetuses with complete perinatal follow‐up. To this end, the following exclusion criteria were applied: (a) non‐viable pregnancies at the time of ultrasound examination; (b) late‐dating ultrasound scans (> 24 weeks); (c) multiple gestations; (d) the presence of fetal chromosomal anomalies or other genetic disorders; (e) fetal structural anomalies; (f) fetal weight below the 10th percentile or above the 90th percentile; (g) oligohydramnios or polyhydramnios; and (h) incomplete perinatal follow‐up.

Linear regression analysis was conducted to generate predicted FEL values for each gestational age interval, including the 5th, 25th, 50th, 75th, 90th, and 95th percentiles with their corresponding 95% confidence intervals (CI). These values were used to create the final nomogram percentiles. A graph of standardized residuals was obtained for this linear regression plot.

Intraobserver reliability was assessed by having four sonologists perform three FEL measurements on 159 fetuses. Interobserver reliability was evaluated by five sonologists, each working in pairs, across a total of 37 fetuses. Intraclass correlation coefficients were calculated using a two‐factor random‐effects model for absolute agreement, accounting for both individual and measurement effects.

To evaluate FEL as a marker for chromosomal anomalies in the entire study population, a multivariate logistic regression model was applied using data from 1265 fetuses. A preliminary bivariate analysis assessed unadjusted associations with potential predictors, including FEL at or below the 5th percentile, ethnicity, maternal age, and aneuploidy screening results. Predictors showing statistical significance at *p* ≤ 0.100 were included in the full predictive model, followed by backward stepwise analysis to derive the adjusted predictive model. Statistical significance was set at *p* < 0.05 (two‐tailed). All data were analyzed using SPSS for Windows, version 27.0.

## 3 Results

**FIGURE 2:**
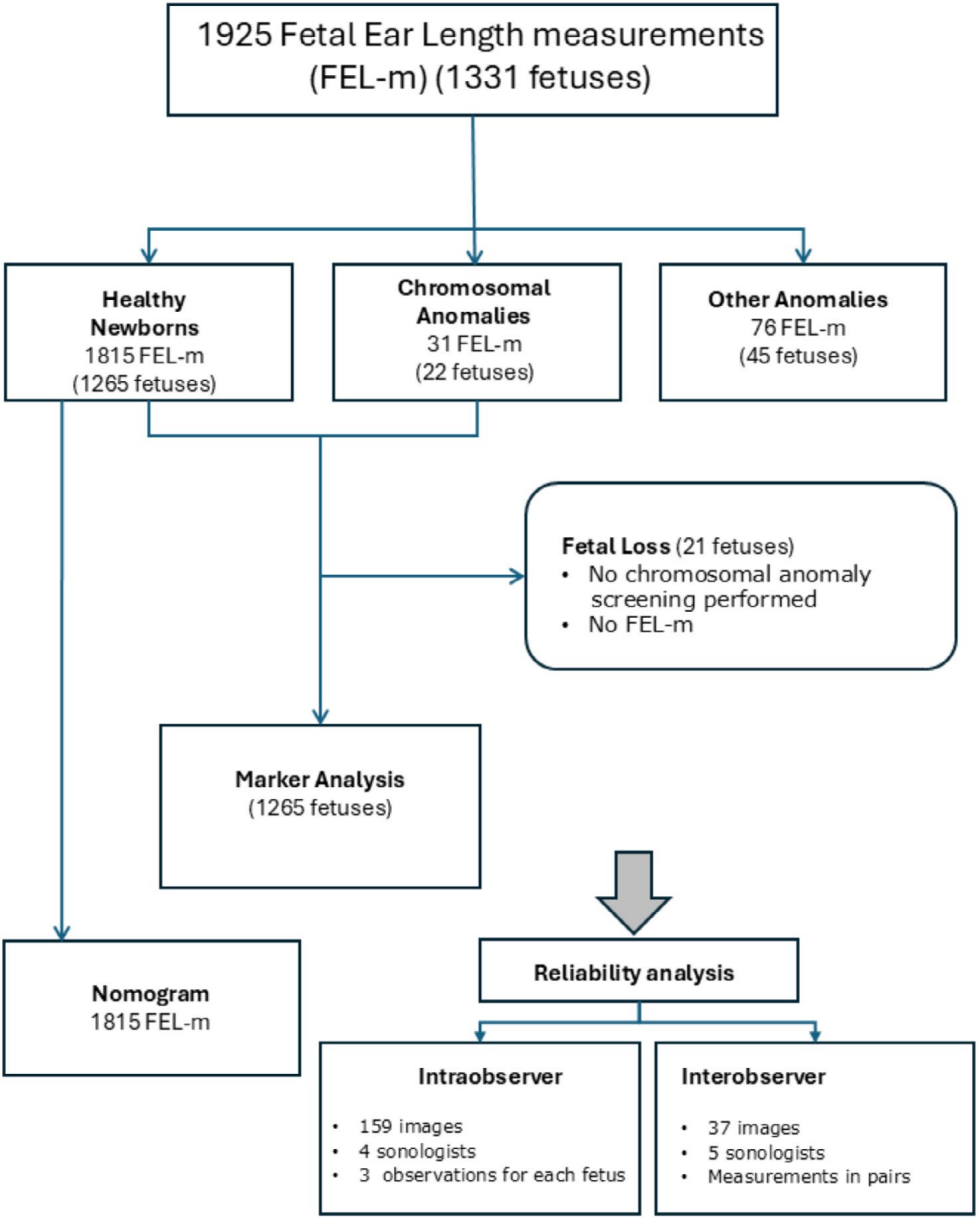
Flowchart of gestations and measurements.

This study included 1331 fetuses with a total of 1925 FEL measurements (Figure 2). The mean maternal age was 31.4 years (16–46 years), with 83.8% of participants identifying as Caucasian, followed by Maghrebi (9.1%), Black (5.6%), and Asian (1.5%) ethnicities (Table 1). Participants originated from 51 different countries, with the majority (65%) being Spanish.

**TABLE 1:** Women and pregnancy characteristics.

Variable	Healthy newborns *N* = 1243	Chromosomal anomalies *N* = 21	Total *N* = 1264	*p*
Maternal age	31.4 (5.7)	35.5 (7.0)	31.4 (5.7)	0.005
Ethnicity				0.121
Caucasian	1044 (84.0%)	15 (71.4%)	1059 (83.8%)	
Black	70 (5.6%)	1 (4.8%)	71 (5.6%)	
Maghrebi	110 (8.8%)	5 (23.8%)	115 (9.1%)	
Asian	19 (1.5%)	0 (0.0%)	19 (1.5%)	
Estimated risks at aneuploidy screening (combined or quadruple test)				< 0.001
Low risk	1102 (88.7%)	3 (14.3%)	1105 (87.4%)	
Intermediate	95 (7.6%)	4 (19.0%)	99 (7.8%)	
High risk	46 (3.7%)	14 (66.7%)	60 (4.7%)	

*Note*: Values in red indicate statistically significant differences between groups (*p* < 0.05).

FEL measurements were successfully obtained in under 3 min in most cases. The distribution by trimester was as follows: first trimester (11–13 + 6 weeks), 899 measurements (46.7%); second trimester (14–24 weeks), 670 measurements (34.8%); and third trimester (24 + 1–34 + 6 weeks), 356 measurements (18.5%). The time spent on the examination did not exceed 3 min in any case. Of the 1925 measurements, only one ear could be assessed in 212 cases (11%), while in just one case (0.05%)—a fetus presenting with severe hydrops due to trisomy 18—neither ear could be evaluated

Among the 1331 pregnancies, 1254 (94.2%) underwent FTS; 52 (3.9%) underwent second‐trimester biochemical screening; 25 (1.9%) did not undergo screening due to maternal preference or late booking; three underwent cfDNA testing due to a history of previous chromosomal anomalies; two required invasive testing due to ultrasound‐detected anomalies; and one underwent invasive testing due to a previous chromosomal anomaly (15q13.3 duplication).

Among the 1331 pregnancies, 1265 resulted in a healthy newborn. A total of 21 fetuses were diagnosed with chromosomal anomalies, five had monogenic disorders, and 36 presented structural anomalies without chromosomal abnormalities. There were four stillbirths: two in the second trimester due to chorioamnionitis and two in the third trimester, one caused by cytomegalovirus infection and the other by placental abruption in a normally inserted placenta.

The chromosomal anomalies diagnosed included 11 trisomies 21, five trisomies 18, two trisomies 13, one trisomy 16, one triploidy, and one 7q22.2‐q31.1 deletion. Additionally, one 22q11.21 microduplication and four monogenic disorders were identified: CHARGE syndrome, Beckwith–Wiedemann syndrome, Meckel–Gruber syndrome, and mevalonate kinase deficiency (Table S1).

**FIGURE 3:**
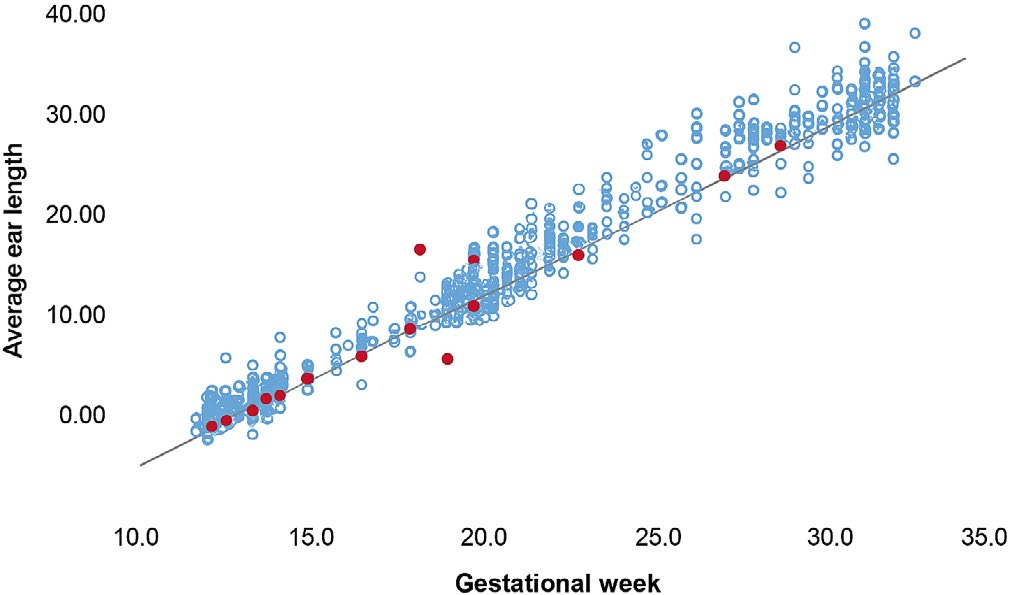
Linear regression between ear length and gestational age: fetuses with chromosomal anomalies are plotted in red.

**FIGURE 4:**
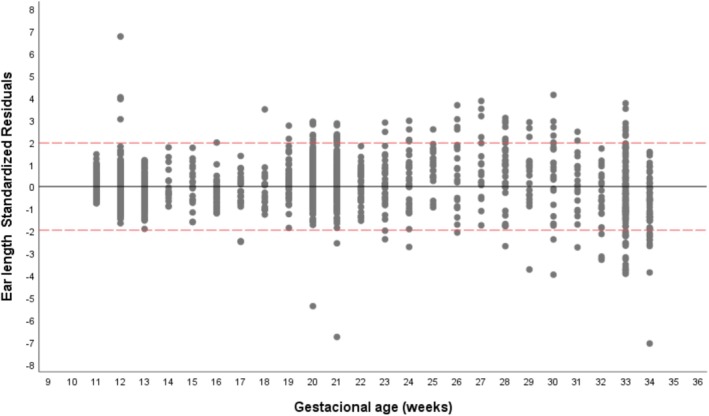
Standardized residuals for linear regression between ear length and gestational age.

A total of 1815 FEL measurements from 1265 healthy fetuses were used to construct a nomogram based on gestational age (Figure 2). The regression plot was calculated as *y* = −8.45 + 1.17*x*, with an *R* ^2^ value of 0.96, indicating that 96% of the variability in the predicted values was explained by gestational age (Figure 3). The standardized residuals of this regression line were evaluated (Figure 4). This dot plot shows that residuals slightly increase with gestational age, a fact already described by other authors. Using this regression model, the predicted mean FEL value for each gestational week was obtained (Table 2), and the nomogram percentiles were subsequently constructed (Table 3).

**TABLE 2:** Descriptive analysis of the predicted values for fetal ear length for each gestation week interval.

Gestational age (weeks)	Sample size (*N*)	Minimum length (mm)	Maximum length (mm)	Mean length (mm)	Standard deviation (mm)
11	145	4.70	5.17	5.07	0.14
12	524	5.64	6.34	5.99	0.23
13	184	6.82	7.52	7.04	0.22
14	16	7.99	8.70	8.20	0.21
15	16	9.17	9.64	9.39	0.17
16	35	10.34	11.04	10.61	0.21
17	18	11.51	11.98	11.66	0.17
18	15	12.69	13.39	13.17	0.21
19	50	13.86	14.57	14.32	0.25
20	261	15.04	15.74	15.37	0.24
21	160	16.21	16.92	16.50	0.23
22	40	17.39	18.09	17.68	0.23
23	28	18.56	19.26	18.91	0.22
24	25	19.73	20.32	20.03	0.21
25	18	20.91	21.61	21.20	0.24
26	20	22.08	22.79	22.41	0.28
27	16	23.26	23.96	23.61	0.21
28	29	24.43	25.14	24.83	0.23
29	17	25.61	26.31	25.90	0.27
30	20	26.78	27.49	27.10	0.22
31	16	27.96	28.66	28.35	0.23
32	21	29.13	29.84	29.48	0.24
33	85	30.31	31.01	30.76	0.19
34	56	31.48	32.19	31.76	0.23

*Note*: The predicted values were calculated from the regression line y = −8.45 + 1.17*fetal ear length.

**TABLE 3:** Nomogram of fetal ear length percentiles (and 95% confidence intervals) by gestational age.

Gestation week	*n*	P5	P10	P25	P50	P75	P90	P95
11	145	4.7 (4.7–4.8)	4.8 (4.7–4.9)	5.1 (4.9–5.1)	5.2 (5.1–5.2)	5.2 (5.2–5.2)	5.2 (5.2–5.2)	5.2 (5.2–5.2)
12	524	5.6 (5.6–5.6)	5.6 (5.6–5.6)	5.8 (5.8–5.9)	6.0 (6.0–6.0)	6.2 (6.1–6.2)	6.3 (6.3–6.3)	6.3 (6.3–6.3)
13	184	6.8 (6.8–6.8)	6.8 (6.8–6.8)	6.8 (6.8–6.9)	6.9 (6.9–7.1)	7.2 (7.2–7.3)	7.4 (7.3–7.5)	7.5 (7.4–7.5)
14	16	8.0 (8.0–8.0)	8.0 (8.0–8.1)	8.0 (8.0–8.1)	8.2 (8.0–8.3)	8.3 (8.2–8.7)	8.6 (8.2–8.7)	8.7 (8.3–8.7)
15	16	9.2 (9.2–9.2)	9.2 (9.2–9.3)	9.2 (9.2–9.4)	9.4 (9.2–9.5)	9.5 (9.4–9.6)	9.6 (9.5–9.6)	9.6 (9.5–9.6)
16	35	10.3 (10.3–10.3)	10.3 (10.3–10.4)	10.5 (10.3–10.6)	10.6 (10.5–10.7)	10.7 (10.6–10.9)	11.0 (10.7–11.0)	11.0 (10.9–11.0)
17	18	11.5 (11.5–11.5)	11.5 (11.5–11.5)	11.5 (11.5–11.6)	11.6 (11.5–11.7)	11.8 (11.6–12.0)	12.0 (11.7–12.0)	12.0 (11.9–12.0)
18	15	12.7 (12.7–13.0)	12.8 (12.7–13.1)	13.0 (12.7–13.2)	13.2 (13.1–13.4)	13.4 (13.2–13.4)	13.4 (13.3–13.4)	13.4 (13.4–13.4)
19	50	13.9 (13.9–14.0)	13.9 (13.9–14.0)	14.1 (14.0–14.3)	14.3 (14.3–14.5)	14.6 (14.4–14.6)	14.6 (14.6–14.6)	14.6 (14.6–14.6)
20	261	15.0 (15.0–15.0)	15.0 (15.0–15.0)	15.2 (15.2–15.2)	15.4 (15.3–15.4)	15.6 (15.5–15.6)	15.7 (15.6–15.7)	15.7 (15.7–15.7)
21	160	16.2 (16.2–16.2)	16.2 (16.2–16.2)	16.3 (16.2–16.3)	16.4 (16.4–16.6)	16.7 (16.6–16.7)	16.9 (16.8–16.9)	16.9 (16.9–16.9)
22	40	17.4 (17.4–17.4)	17.4 (17.4–17.5)	17.5 (17.4–17.5)	17.6 (17.5–17.7)	17.9 (17.7–18.1)	18.1 (17.9–18.1)	18.1 (18.0–18.1)
23	28	18.6 (18.6–18.7)	18.6 (18.6–18.7)	18.7 (18.6–18.8)	18.9 (18.8–19.0)	19.1 (19.0–19.1)	19.2 (19.1–19.3)	19.3 (19.1–19.3)
24	25	19.7 (19.7–19.7)	19.7 (19.7–19.9)	19.9 (19.7–20.0)	20.1 (19.9–20.1)	20.2 (20.1–20.3)	20.3 (20.2–20.3)	20.3 (20.3–20.3)
25	18	20.9 (20.9–20.9)	20.9 (20.9–21.0)	21.0 (20.9–21.2)	21.3 (21.0–21.3)	21.4 (21.3–21.6)	21.6 (21.4–21.6)	21.6 (21.4–21.6)
26	20	22.1 (22.1–22.1)	22.1 (22.1–22.2)	22.1 (22.1–22.3)	22.4 (22.2–22.7)	22.7 (22.5–22.8)	22.8 (22.7–22.8)	22.8 (22.7–22.8)
27	16	23.3 (23.3–23.4)	23.3 (23.3–23.5)	23.5 (23.3–23.6)	23.6 (23.5–23.7)	23.7 (23.6–24.0)	24.0 (23.7–24.0)	24.0 (23.8–24.0)
28	29	24.4 (24.4–24.6)	24.6 (24.4–24.7)	24.7 (24.6–24.7)	24.9 (24.7–25.0)	25.0 (24.9–25.1)	25.1 (25.0–25.1)	25.1 (25.1–25.1)
29	17	25.6 (25.6–25.6)	25.6 (25.6–25.7)	25.7 (25.6–25.7)	25.7 (25.7–26.1)	26.1 (25.8–26.3)	26.3 (26.1–26.3)	26.3 (26.2–26.3)
30	20	26.8 (26.8–26.9)	26.8 (26.8–26.9)	26.9 (26.8–27.0)	27.1 (26.9–27.3)	27.3 (27.1–27.4)	27.4 (27.3–27.5)	27.5 (27.4–27.5)
31	16	28.0 (28.0–28.1)	28.0 (28.0–28.2)	28.2 (28.0–28.3)	28.3 (28.2–28.5)	28.5 (28.3–28.7)	28.7 (28.5–28.7)	28.7 (28.6–28.7)
32	21	29.1 (29.1–29.2)	29.2 (29.1–29.2)	29.2 (29.1–29.4)	29.5 (29.2–29.6)	29.7 (29.5–29.8)	29.8 (29.6–29.8)	29.8 (29.8–29.8)
33	85	30.3 (30.3–30.4)	30.4 (30.3–30.5)	30.7 (30.5–30.8)	30.8 (30.8–30.9)	30.9 (30.9–30.9)	31.0 (30.9–31.0)	31.0 (31.0–31.0)
34	56	31.5 (31.5–31.5)	31.5 (31.5–31.5)	31.6 (31.5–31.6)	31.7 (31.6–31.8)	32.0 (31.8–32.1)	32.1 (32.1–32.2)	32.2 (32.1–32.2)

The intraobserver intraclass correlation coefficient (ICC) was 0.996 (95% CI: 0.995–0.997), indicating excellent reliability. Similarly, the interobserver ICC was 0.998 (95% CI: 0.978–0.999), also demonstrating outstanding reliability.

In the entire study population, the mean gestational age at FEL measurement was similar between fetuses with normal perinatal outcomes (19 + 6 weeks) and those diagnosed with chromosomal anomalies (19 + 2 weeks). No significant differences in maternal age or ethnicity were observed between the two groups. For any gestational age, FEL was significantly shorter in fetuses with chromosomal anomalies than in those with normal outcomes (10.07 vs. 13.6 mm; *p* = 0.043; 95% CI: 0.09–5.89). A total of 50.1% of healthy fetuses had an FEL ≤ 5th percentile, compared with 81.0% of fetuses with chromosomal anomalies (*p* = 0.005). The sensitivity and specificity of FEL as a predictive marker of chromosomal anomalies were 81.0% and 49.9%, respectively.

Unadjusted analysis revealed a significant association between chromosomal anomalies and several factors, including maternal age, aneuploidy screening results, and FEL ≤ 5th percentile. The final multivariate model indicated that, after adjusting for aneuploidy screening results, FEL values ≤ 5th percentile were associated with an increased risk of chromosomal anomalies (OR = 3.15). However, the 95% CI (0.99–9.96) suggests limited precision, as this interval contains the null value “1” (*p* = 0.051) (Table 4).

**TABLE 4:** Steps to obtain the adjusted predicted model in a multivariate logistic regression analysis (*N* = 1264): (A) Unadjusted association between each candidate predictor and outcome, (B) Full predicted model with candidate variables, and (C) final adjusted predicted model.

Unadjusted analysis	Beta	OR (95% CI)	*p*
Maternal age	0.120	1.13 (1.04; 1.23)	0.005
Ethnicity			
Caucasian		Reference	0.218
Black	−0.006	0.99 (0.13; 7.64)	0.996
Maghrebi	1.152	3.16 (1.13; 8.87)	0.029
Asian	−16.96	—	— (*)
Risk at aneuploidy screening			
Low		Reference	
Intermediate	2.739	15.47 (3.41; 70.12)	< 0.001
High	4.717	111.8 (31.04; 402.6)	< 0.001
FEL ≤ 5th percentile	1.442	4.23 (1.42; 12.64)	0.010

Full predicted model	Beta	OR (95% CI)	*p*
Maternal age	0.007	1.01 (0.90; 1.09)	0.877
Ethnicity			
Caucasian		Reference	
Black	−0.425	0.65 (0.07; 6.07)	0.709
Maghrebi	0.602	1.83 (0.56; 6.0)	0.322
Asian	−16.516	—	—
Aneuploidy screening			
Low		reference	
Intermediate	2.674	14.5 (3.0; 69.8)	0.001
High	4.519	91.8 (23.8; 354.5)	< 0.001
≤ 5th percentile	1.121	3.10 (1.0; 9.8)	0.059
Constant	−6.850		

Final adjusted predicted model	Beta	OR (95% CI)	*p*
Combined test			
Low	Reference		
Intermediate	2.730	15.3 (3.4; 69.8)	< 0.001
High	4.600	99.4 (27.4; 360.7)	< 0.001
≤ 5th percentile	1.147	3.15 (0.99; 9.96)	0.051
Constant	−6.632		

*Note*: Values in red indicate statistically significant differences between groups (*p* < 0.05).

## 4 Discussion

### 4.1 Main Findings

This study yielded three principal findings: First, a nomogram for FEL was developed based on 1815 measurements obtained from 1265 pregnancies along gestation within the general pregnant population of Catalonia. Second, the study demonstrated outstanding reliability, as evidenced by excellent intraobserver and interobserver intraclass correlation coefficients. Third, significant differences in FEL were identified between fetuses with a normal outcome and those diagnosed with chromosomal anomalies.

### 4.2 Comparison With the Literature

#### 4.2.1 FEL Nomogram

Previous research has constructed FEL nomograms based on gestational age, with sample sizes ranging from 124 to 2583 cases across various studies (Shimizu et al. 1992; Chitkara et al. 2000; Sacchini et al. 2003; Yeo et al. 2003; Joshi et al. 2011; Hatanaka et al. 2011; Özdemir et al. 2014). These studies consistently demonstrated a linear correlation between gestational age and FEL. Notably, only one of these studies, which included 450 fetuses, focused specifically on the first trimester of pregnancy (Sacchini et al. 2003) (Table 5).

**TABLE 5:** Reported nomograms of fetal ear length.

Author	Sample size (*N*)	Gestational period (weeks)	2D or 3D ultrasound
Shimizu et al. (1992)	124	18–42	2D
Chitkara et al. (2000)	2583	15–40	2D
Sacchini et al. (2003)	450	11–14	2D
Yeo et al. (2003)	447	14–41	2D
Joshi et al. (2011)	787	18–22	2D
Hatanaka et al. (2011)	114	19–23.6	3D
Özdemir et al. (2014)	389	16–28	2D

Our investigation incorporated 1925 measurements from 1332 fetuses spanning gestational ages from 11 + 2 to 33 + 6 weeks. Among these, 899 measurements were conducted during the first trimester, distinguishing this study as the largest one targeting the Southern European population for FEL nomogram development.

#### 4.2.2 FEL Efficacy as an Aneuploidy Marker

Several studies have investigated the role of ultrasound measurement of FEL in predicting fetal aneuploidy, with some yielding significant results. Five studies conducted between 14 and 38 weeks of gestation reported sensitivities ranging from 32% to 80% when using FEL as a marker of chromosomal anomalies (Yeo et al. 2003; Awwad et al. 1994; Shimizu et al. 1997; Lettieri et al. 1993; Chitkara et al. 2002). However, in other studies, including the single first‐trimester series, FEL was not found to be a reliable predictor (Sacchini et al. 2003; Witkowski et al. 2023) (Table 6).

**TABLE 6:** Reported studies on the use of fetal ear length in the prediction of chromosomal anomalies.

Author	*n*	Gestational period (weeks)	Aneuploidy risk	FEL percentile	Chromosomal anomalies (*N*)	Sensitivity	Specificity	Positive predictive value	Negative predictive value
Awwad et al. (1994)	418	20–28	Low risk		10	75	99	8.5	
Shimizu et al. (1997)	29	18–38	High risk	5th	10	80	84	73	89
Lettieri et al. (1993)	424	14–25	High risk	10th	15	71	92	23	99
Chitkara et al. (2002)	1311	16–36	High risk	10th	37	32	93	14	98
Yeo et al. (2003)	447	14–41	High risk	10th	96	66	41% trisomy 21, 93% trisomy 18, 100% trisomy 13		
Sacchini et al. (2003)	450	11–14	High risk	5th	41		NS		
Witkowski et al. (2023)	147	17–39 + 5	Low risk	5th	15		NS		
Roosen et al. (2024)	100	20–37	High risk		50		Significant differences in ear length and width, and ear surface between healthy and chromosomally abnormal fetuses.		

*Note*: NS, not significant.

The most extensive study assessing the utility of FEL as a predictor of aneuploidies was conducted in high‐risk fetuses (Chitkara et al. 2002). Researchers analyzed 1311 singleton pregnancies between 16 and 35 weeks of gestation, all of which underwent genetic amniocentesis in the second or third trimester. A short ear was defined as an FEL ≤ 10th percentile for gestational age. Among the 34 fetuses diagnosed with significant chromosomal anomalies, 11 (32%) presented with short ears. In six cases (18%), the short ear was the only detectable marker via ultrasound. The combination of a short ear and other abnormal ultrasound findings increased the positive predictive value (PPV) to 46%, compared with that of a short ear (14%), or abnormal ultrasound alone (11%). The authors concluded that FEL measurement, either alone or in combination with other ultrasound markers, may be useful in predicting aneuploidies in high‐risk pregnancies. However, they noted its limited sensitivity and emphasized the need for further investigation regarding its utility in low‐risk populations.

The only study conducted in the first trimester assessed FEL in 450 high‐risk pregnancies between 11 and 14 weeks before chorionic villus sampling for karyotyping (Sacchini et al. 2003). Among fetuses with trisomy 21, ear length was significantly shorter compared with normal fetuses, with an average deviation of 0.45 mm below the expected mean (*p* = 0.013). However, only 6.3% of trisomy 21 cases had ear lengths below the 5th percentile of the normal range. As a result, the authors concluded that, while the difference was statistically significant, it was not pronounced enough to serve as a reliable marker in aneuploidy screening.

Recently, a study using 3D ultrasound to assess ear length, width, and surface area reported that fetuses with trisomy 18 had ears that were, on average, 0.423 cm shorter than those of unaffected fetuses (*p* < 0.001). Additionally, the rate of ear growth per week of gestation was 0.046 cm lower in fetuses with trisomy 18 than in controls (*p* = 0.027). In trisomy 21 and other syndromes, no significant differences in ear length were observed. However, variations in ear morphology were noted, highlighting the advantages of 3D ultrasound as a complementary technique to traditional 2D ultrasound (Roosen et al. 2024).

### 4.3 Strengths and Limitations

A strength of our study is that it includes the largest sample development of a nomogram specifically tailored to the Southern European population for FEL. It includes the largest dataset of first‐trimester measurements, effectively demonstrating the feasibility of assessing fetal ears using 2D ultrasound during this early gestational period while also evaluating intra‐ and interobserver reliability, with excellent intraclass correlation coefficients. Furthermore, our study identifies a significant association between FEL and chromosomal anomalies, underscoring its potential utility in the prenatal diagnosis of aneuploidies.

However, our study was not without limitations. Since the study cohort comprised a low‐risk population, the number of fetuses diagnosed with chromosomal anomalies was limited, which may decrease the accuracy of the estimates. Furthermore, while the data suggest an association between FEL measurements ≤ 5th percentile and the occurrence of chromosomal anomalies (odds ratio, OR = 3.15), the wide 95% confidence interval for this association underscores the uncertainty of this estimate. Larger studies that include more cases of fetal anomalies are needed to confirm these findings.

## 5 Conclusion

Importantly, this study demonstrates the feasibility of assessing fetal ear length using 2D ultrasound, even in the first trimester of pregnancy. This finding supports further research into additional fetal ear parameters to refine and enhance the prenatal prediction of chromosomal anomalies.

## Author Contributions

**Elisabet Baldrich:** principal investigator and doctoral candidate, responsible for conceptualization, methodology, data collection, formal analysis, and original draft preparation. **Montse Comas, Alicia Maldonado, and Marcos Blasco:** contributed to data collection and participated in manuscript review and editing. **Maria Iniguez‐Cruz and Josep‐Maria Manresa‐Domínguez:** performed statistical analysis and participated in manuscript review and editing. **Antoni Borrell:** thesis director, provided supervision, validation, and contributed to manuscript review and editing. All authors have read and approved the final version of the manuscript.

## Ethics Statement

The study protocol was approved by the Research Ethics Committees of IDIAP‐Jordi Gol (CEIm code: 22/040‐P) and by Parc Tauli Health Corporation (Ref.2022/6059).

## Consent

The principles of the Declaration of Helsinki were followed. The study did not involve any discomfort or risk for the pregnant participants, as it did not alter standard clinical management and only slightly increased the duration of the ultrasound examination. All data were anonymized, ensuring the confidentiality of electronic health records in accordance with national and international legislation. Regarding personal data protection, the study complies with the requirements of the European Union General Data Protection Regulation (Regulation (EU) 2016/679 of the European Parliament and of the Council of April 27, 2016), in force since May 25, 2016, and applicable from May 24, 2018. All participants were provided with an informational document about the study and an informed consent form, which was signed by both the patient and the investigator.

## Conflicts of Interest

The authors declare no conflicts of interest.

## Supporting information


**Table S1:** Chromosomal and other genetic anomalies detected in our study population.

## Data Availability

The data that support the findings of this study are available on request from the corresponding author. The data are not publicly available due to privacy or ethical restrictions.
