# Supplementary material for: Efficacy of Fetal Ear Length as a Prenatal Marker of Chromosomal Anomalies: A Prospective, Multicenter Cohort Study in a Southern European Population
Source: J Clin Ultrasound. 2025 Aug 14;54(1):88–98. doi: 10.1002/jcu.70040 (PMC12766865; doi:10.1002/jcu.70040)
Supplement: Supplementary file 1 — Table S1: Chromosomal and other genetic anomalies detected in our study population. [file JCU-54-88-s001.docx]

**Supplementary table 1.** Chromosomal and other genetic anomalies detected in our study population.

| Case No. | Gestational Age  (weeks) | Risk at the Combined Test | NT (mm) | Fetal Structural Anomalies | Second-trimester markers | Genetic Anomaly | Median ear length (mm) | Percentile | Perinatal Outcome |
| --- | --- | --- | --- | --- | --- | --- | --- | --- | --- |
| 1 | 11.3 | High | 1.5 |  |  | Trisomy 21 | 5.0 | 25 th | TOP, does not accept autopsy |
| 2 | 11.5 | High | 3.7 |  | Reversed a-wave in DV flow | Trisomy 21 | 3.85 | <5th | TOP, confirmed in autopsy |
| 3 | 11.5 | High | 2.3 |  |  | Trisomy 21 | 5.6 | 10th | TOP, Confirmed in autopsy |
| 4 | 11.6 | High | 6.2 |  | Absent NB, reversed a-wave DV flow | Trisomy 21 | 4.46 | <5th | TOP, does not accept autopsy |
| 5 | 12.1 | High | 1.45 |  |  | Trisomy 21 | 5.75 | 15th | TOP, Confirmed in autopsy |
| 6 | 12.4 | High | 2.2 |  |  | Trisomy 21 | 5.05 | <5th | TOP, Confirmed in autopsy |
| 7 | 12.6 | High | 2.7 | Atrioventricular canal defect |  | Trisomy 21 | 5.45 | <5th | Confirmed in newborn |
| 8 | 13.0 | High | 3.7 |  | Absent NB, reversed a-wave in DV flow | Trisomy 21 | 5.35 | <5th | Confirmed in newborn |
| 9 | 15.1 | High | 1 | Perimembranous VSD |  | Trisomy 21 | 5.45 | <5th | TOP, confirmed in autopsy |
| 10 | 20.6 | High | 1 |  |  | Trisomy 21 | 14.35 | <5th | Confirmed in newborn |
| 11 | 31.6 | High | 5 | Perimembranous VSD | Absent NB | Trisomy 21 | 27.5 | <5th | Confirmed in newborn |
| 12 | 13.4 | Intermediate | 2 | Fetal tachycardia and 2 vessel umbilical cord |  | Trisomy 13 | 6.75 | <5th | TOP, confirmed autopsy |
| 13 | 18.6 | Low | - | Polymalformed |  | Trisomy 13 | 11.85 | <5th | TOP, does not accept autopsy |
| 14 | 11.4 | Low | 1.2 | Holoprosencephaly |  | Trisomy 18 | 5.65 | 50th | TOP, confirmed in autopsy |
| 15 | 12.4 | Low | 1.3 | Polymalformed |  | Trisomy 18 | 4.85 | <5th | TOP, confirmed in autopsy |
| 16 | 13.5 | High | 7.1 | Fetal hydrops and omphalocele |  | Trisomy 18 | 5.7 | <5th | TOP, confirmed in autopsy |
| 17 | 15.3 | High | 1.7 |  |  | Trisomy 18 | 6.9 | <5th | TOP, confirmed in autopsy |
| 18 | 17 | Intermediate | 1.5 | VSD and omphalocele | Bilateral choroid plexus cysts | Trisomy 18 | 7.45 | <5th | TOP, confirmed in autopsy |
| 19 | 11.6 | high | 1.2 |  |  | Trisomy 16 mosaic 50% | 4.6 | <5th | TOP, confirmed in autopsy |
| 20 | 12.3 | High | 1.1 | Holoprosencephaly | Absent NB | Triploidy | 4.9 | <5th | TOP, does not accept autopsy |
| 21 | 13.3 | Intermediate | 1.8 |  |  | Deletion 7q22.2q31.1 | 5.7 | <5th | Confirmed in newborn |
| 22 | 30.5 | Low | 1.4 | Right aortic arch | ALSA | Duplication 22q11.21 | 24.5 | <5th | Confirmed in newborn |
| 23 | 13.0 | Low | 1.4 |  | Reversed a-wave in DV flow | Meckel Gruber syndrome | 5.95 | <5th | TOP, confirmed in autopsy |
| 24 | 12.2 | Low | 1.9 | Right hand polydactyly |  | CHARGE syndrome | 5.25 | <5th | Confirmed in newborn |
| 25 | 12.5 | Low | 1.5 |  |  | Beckwith-Wiedemann syndrome | 6 | <5th | Confirmed in newborn |
| 26 | 13.6 | Low | 2.5 | Fetal growth restriction and polymalformed |  | Mevalonate kinase deficiency. | 8.5 | 75th | Confirmed in newborn |

NT, nuchal translucency; TOP, termination of pregnancy; NB, nasal bone; DV, ductus venosus; VSD, ventricular septal defect; ALSA, aberrant left subclavian artery.
